# Interleukin-6 is the strongest predictor of 30-day mortality in patients with cardiogenic shock due to myocardial infarction

**DOI:** 10.1186/cc11467

**Published:** 2012-08-13

**Authors:** René P Andrié, Ulrich M Becher, Ricarda Frommold, Vedat Tiyerili, Jan W Schrickel, Georg Nickenig, Jörg O Schwab

**Affiliations:** 1 Department of Cardiology, University of Bonn, Sigmund-Freud-Str. 25, 53105 Bonn, Germany

## Abstract

**Introduction:**

Cardiogenic shock (CS) remains the leading cause of death in patients hospitalized for myocardial infarction (MI). Systemic inflammation with inappropriate vasodilatation is observed in many patients with CS and may contribute to an excess mortality rate. The purpose of this study was to determine the predictive role of serial measurements of Nt-proBNP, interleukin-6 (IL-6), and procalcitonin (PCT) for 30-day mortality in patients with CS due to MI.

**Methods:**

The present study is a prospective single-center study including 87 patients with MI complicated by CS treated with acute revascularization and intraaortic balloon counterpulsation (IABP) support. Predictive values of plasma levels at admission (T_0_), after 24 hours (T_1_), and after 72 hours (T_2_) were examined according to 30-day mortality.

**Results:**

Significant differences between survivors (*n *= 59) and nonsurvivors (*n *= 28) were seen for Nt-proBNP at T_0_, for IL-6 at T_0 _and T_1_, and for PCT at T_1 _and T_2_. According to ROC analyses, the highest accuracy predicting 30-day mortality was seen at T_0 _for IL-6, at T_1 _for PCT, and at T_2 _for PCT. In univariate analysis, significant values were found for Nt-proBNP at T_1_, and for IL-6 and PCT at all points in time. Within the multivariate analysis, age, creatinine, and IL-6 were significant determinants of 30-day mortality, in which IL-6 showed the highest level of significance.

**Conclusions:**

In patients with MI complicated by CS, IL-6 represented a reliable independent early prognostic marker of 30-day mortality. PCT revealed a significant value at later points in time, whereas Nt-proBNP seemed to be of lower relevance.

## Introduction

Cardiogenic shock (CS) complicating acute myocardial infarction (AMI) occurs in 5% to 10% of hospitalized patients and is the leading cause of intrahospital mortality after AMI [1,2]. Early revascularization of the infarct-related artery is the fundamental step in therapeutic strategies and has been shown to improve long-term survival in patients with CS [3,4]. However, some patients fail to show clinical and hemodynamic improvement, with a poor prognosis despite successful immediate revascularization.

In the classic pathophysiologic view, CS is the result of temporary and permanent disorders in the circulatory system [5]. New irreversible injury, reversible ischemia, and damage from prior infarction contribute to left ventricular dysfunction. N-terminal-pro-B-type natriuretic peptide (Nt-proBNP) is used for the early diagnosis of heart failure (HF) in patients with acute dyspnea [6]. In patients with chronic HF and AMI, Nt-proBNP is a reliable predictor of increased mortality [7]. In addition, elevated BNP levels in patients with septic shock are indicative of septic cardiomyopathy [8].

Systemic inflammation with inappropriate vasodilatation, as evidenced by a normal to low range of systemic vascular resistance, is observed in many patients with CS and may contribute to an excess mortality rate [9]. Accordingly, it has been postulated that CS causes a systemic inflammatory response syndrome (SIRS) by the release of pro-inflammatory mediators like interleukin-6 (IL-6) and tumor necrosis factor alpha (TNF-α) [5]. Signs of systemic inflammation such as fever, leukocytosis, and elevated acute-phase reactants are frequently observed in patients with AMI and CS. High levels of systemic inflammation seem to be associated with impaired survival despite early revascularization [5]. It has been shown that patients with CS and multiorgan failure (MOF) exhibit concentrations of IL-6 of the same magnitude as do patients with septic shock [10,11].

Procalcitonin (PCT) is a well-established biomarker for the diagnosis of sepsis [12]. PCT reflects the severity of bacterial infection and is used to monitor progression of infection into sepsis, severe sepsis, or septic shock. Moreover, PCT is used to measure the activity of the systemic inflammatory response [12]. The increase of PCT in patients with sepsis correlates with mortality [12]. A limited number of studies have reported that increased levels of PCT are related to AMI [13,14].

In the present study, we analyzed the relation between plasma levels of Nt-proBNP, IL-6, and PCT at different points in time early after admission and 30-day mortality in patients with CS due to AMI, treated with immediate revascularization and IABP support. Moreover, central determinants of organ failure, hemodynamics, and revascularization were analyzed in relation to short-term prognosis and to levels of Nt-proBNP, IL-6, and PCT.

## Materials and methods

### Subjects

The present study is a prospective observational single-center study at a university hospital. Between 2008 and 2010, 87 patients with AMI complicated by CS at admission were included. The study complies with the Declaration of Helsinki and was approved by the local medical ethics committee. Patients or, in case of unconsciousness, relatives signed a consent form. Underlying causes of CS were categorized as ST-elevation myocardial infarction (STEMI) and non-ST-elevation myocardial infarction (NSTEMI). CS was defined by the presence of one of the following criteria: (a) peak systolic pressure <90 mm Hg for >30 minutes after the correction of hypovolemia, hypoxemia, and acidosis or need for vasopressor and/or inotropic therapy; (b) signs of organ hypoperfusion such as oliguria/anuria, changes in mental state, or elevated serum lactate concentrations (>2.0 m*M*). Patients with ongoing cardiopulmonary resuscitation (CPR) at admission were excluded from the study. Further exclusion criteria were age <18 years, immunosuppressive therapy, preexisting infectious diseases, mechanical assist devices other than an intraaortic balloon counterpulsation (IABP), mechanical cardiac complications, and coronary artery bypass grafting (CABG) or any surgery in the last 4 weeks before onset of shock.

All patients were taken directly to the cardiac catheterization laboratory for angiography. All patients had undergone implantation of an IABP (Datascope System CS100 and CS100i, Datascope, Oakland, NJ, USA).

Based on the primary outcome variable all-cause mortality at day 30, analyses were performed to identify differences between survivors and nonsurvivors and predictors of worse outcome.

### Hemodynamic measurements

Stroke volume (SV) and left ventricular ejection fraction (EF) were quantified invasively with levocardiography. If levocardiography could not be performed, EF was quantified with echocardiography. Cardiac output (CO) (L/min) was calculated as SV × heart rate. Cardiac power output (CPO) (W) was calculated as mean arterial pressure (MAP) × CO/451, left ventricular work (LVW) as (MAP - left ventricular end-diastolic pressure (LVEDP)) × CO × 0.0136 (kg-m/min), and systemic vascular resistance (SVR) (dyne s/cm^5^) as 80 × (MAP - central venous pressure (CVP))/CO. Left ventricular work index (LVWI) (kg-m/min × m²), cardiac power index (CPI) (W/m²), and systemic vascular resistance index (SVRI) (dyne s/cm^5^/m²) were computed by substituting CO with CI in the respective formulas. Stroke work (SW) (g-m) was calculated as (MAP - LVEDP) × 0.0136 × SV. Stroke work index (SWI) (g-m/m²) was determined by SW/body surface area. Microcirculatory power index (micPI) was calculated as CI × (MAP-CVP) × Hkt-factor.

### Blood sampling

The first blood sample (T_0_) was taken immediately after admission. The second blood sample (T_1_) was taken after 24 hours, and the third blood sample (T_2_), after 72 hours. Nt-proBNP (Siemens Healthcare Diagnostics GmbH, Munich, Germany), IL-6 (Siemens Healthcare Diagnostics GmbH) and PCT (Roche Diagnostics GmbH, Mannheim, Germany) were measured immediately. Further routine laboratory parameters were quantified by using commercially available assays.

### Organ failure

Acute renal failure was defined as an increase of the serum creatinine concentration >2.5 mg/dl, an increase in creatinine >25% from baseline, a diuresis <500 ml/24 hours, or need for hemofiltration. Estimated glomerular filtration rate (eGFR) was calculated by using the Cockroft and Gault formula. Systemic inflammatory response syndrome (SIRS) was defined by approved criteria [5]. Multiorgan failure (MOF) was defined as presence of two or more organ failures, other than cardiovascular. As scoring system of ICU mortality the APACHE II score was used.

### Statistical analysis

Continuous variables are expressed as mean + standard deviation (SD) if normally distributed and as median (interquartile range) if not normally distributed. Continuous variables were tested for normal distribution with the use of the Kolmogorov-Smirnov test. The Student *t *test was performed for normally distributed continuous variables, and the Mann-Whitney *U *test for nonnormally distributed continuous variables. Testing for homogeneity of variance was performed with the Levene test. Categoric variables are given as frequencies and percentages, and the χ^2 ^test with the Fisher Exact test was used for data analysis. Receiver operating characteristics (ROC) curves were performed to determine cut-off levels of Nt-proBNP, IL-6, and PCT, with the highest sensitivity and specificity predicting 30-day mortality. The cumulative survival plots were estimated with the Kaplan-Meier method. Survival in groups was compared with the log-rank test. Univariate Cox regression analysis was performed to identify significant independent predictors of outcome.

For multivariate analysis, age, CPI, mean dose of catecholamines, lactate, creatinine, and IL-6 or PCT were included. For this analysis, a backward stepwise selection method was used. Results are reported as adjusted hazard ratio (HR) with 95% confidence interval (CI). A two-sided *P *value less 0.05 was considered statistically significant. Statistical analyses were performed by using SPSS software version 18.0 (SPSS, Chicago, IL, USA).

## Results

### Primary end point 30-day mortality

The overall 30-day mortality was 32.2% (*n *= 28 nonsurvivors, *n *= 59 survivors). No patient died in the catheterization laboratory. Six patients died before T_1_, and six further patients died before T_2_. Main cause of death was MOF. Mortalities showed no significant differences in STEMI versus NSTEMI patients, male versus female patients, or patients older than 75 years versus 75 years or younger (data not shown).

### Baseline patient characteristics

Baseline patient characteristics of survivors and nonsurvivors are shown in Table 1. Age, percentage of patients receiving mechanical ventilation, serum lactate levels, hemoglobin, and serum creatinine differed significantly between the nonsurvivor and survivor groups. There were no further differences concerning patient characteristics and demographic data.

**Table 1:** Baseline characteristics of study population according to outcome

	All patients(*n *= 87)	Survivors(*n *= 59)	Nonsurvivors(*n *= 28)	*P *value
Age (years)	67.78 ± 13.4	64.68 ± 14.3	74.32 ± 8.4	<0.0001
Age >75 years (%)	35 (40.2)	19 (32.2)	14 (50.0)	0.156
Male (%)	68 (78.2)	46 (78.0)	22 (78.6)	0.949
STEMI/NSTEMI (%)	44/43 (50.6/49.4)	30/29 (50.8/49.2)	14/14 (50.0/50.0)	0.941
Diabetes (%)	29 (33.3)	21 (35.6)	8 (28.6)	0.629
Hypertension (%)	53 (60.9)	37 (62.7)	16 (57.1)	0.645
Dyslipidemia (%)	43 (49.4)	31 (52.5)	12 (42.9)	0.493
Smoker (%)	31 (35.6)	23 (39.0)	8 (28.6)	0.473
Obesity (%)	20 (23.0)	15 (25.4)	5 (17.9)	0.587
BMI (kg/m^2^)	27.6 ± 5.5	27.7 ± 5.8	27.2 ± 4.8	0.739
Family history (%)	11 (12.6)	7 (11.9)	4 (14.3)	0.741
CAD (%)	42 (48.3)	28 (47.5)	14 (50.0)	0.825
Previous MI (%)	25 (28.7)	17 (28.8)	8 (28.6)	0.981
Previous PCI (%)	24 (27.6)	17 (28.8)	7 (25.0)	0.801
Previous CABG (%)	6 (6.9)	4 (6.8)	2 (7.1)	0.950
Chronic heart failure (%)	11 (12.6)	8 (13.6)	3 (10.7)	0.978
Previous stroke (%)	10 (11.5)	8 (13.6)	2 (7.1)	0.490
Chronic renal failure (%)	10 (11.5)	5 (8.5)	5 (17.9)	0.281
Mechanical ventilation at admission (%)	43 (49.4)	28 (47.5)	15 (53.6)	0.651
Mechanical ventilation at any time (%)	62 (71.3)	36 (61.0)	26 (91.3)	0.013
Transferred patients (%)	39 (44.8)	29 (49.2)	10 (35.7)	0.259
Preclinical CPR (%)	23 (26.4)	16 (27.1)	7 (25.0)	0.834
Serum creatinine (mg/dl)	1.44 (1.17/2.10)	1.34 (1.09/1.58)	1.99 (1.45/3.45)	<0.0001
Hemoglobin (g/dl)	12.5 (10.5/14.4)	13.1 (11.1/14.9)	10.8 (10.0/13.5)	0.034
Troponin I (ng/ml)	14.7 (2.9/62.5)	9.1 (1.9/54.4)	21.0 (9.0/77.0)	0.069
Blood glucose (mg/dl)	199.0 ± 129.9	201.3 ± 142.1	193.3 ± 96.5	0.942
Serum lactate (m*M*)	1.9 (1.1/6.0)	1.4 (1.1/3.2)	5.8 (1.6/12.7)	0.008

Values are expressed as *n *(%), or mean ± SD. BMI, body mass index; CABG, coronary artery bypass grafting; CAD, coronary artery disease; CPR, cardiopulmonary resuscitation; MI, myocardial infarction; NSTEMI, non-ST-elevation myocardial infarction; PCI, percutaneous coronary intervention; STEMI, ST-elevation myocardial infarction.

### Diagnostic and therapeutic characteristics

All patients underwent coronary angiography immediately after admission. Diagnostic and therapeutic characteristics are summarized in Table 2. Percutaneous coronary intervention (PCI) was performed technically successfully in 77 (96.3%) patients, defined by a patent vessel with TIMI >2 flow, <50% residual stenosis, and no emergency CABG. Rates of successful PCI were similar in both groups (Table 2). The most common reasons for not attempting revascularization/surgery were a high-risk operation due to low ejection fraction, irreversible MOF, and coronary vessels not suitable for PCI or CABG. Rate of periinterventional CPR was significantly elevated in the nonsurvivor group.

**Table 2:** Procedural characteristics of study population according to outcome

	All patients(*n *= 87)	Survivors(*n *= 59)	Nonsurvivors(*n *= 28)	*P *value
EF (%)	35.2 ± 11.6	34.7 ± 11.6	36.4 ± 11.9	0.548
EF <30% (%)	24 (27.6)	16 (27.1)	9 (32.1)	0.789
Three-vessel disease (%)	51 (58.6)	34 (57.6)	17 (60.7)	0.814
Infarct artery				
Left main (%)	6 (6.9)	4 (6.8)	2 (7.1)	0.950
Left anterior descending (%)	43 (49.4)	29 (49.2)	14 (49.2)	0.941
Right coronary artery (%)	21 (24.1)	13 (22.0)	8 (28.6)	0.691
Left circumflex (%)	24 (27.6)	15 (25.4)	9 (32.1)	0.690
Bypass graft (%)	1 (1.1)	1 (1.7)	0 (0)	0.488
Revascularization procedure (PCI or CABG) (%)	82 (94.3)	57 (96.6)	25 (89.3)	0.323
PCI left main (%)	12 (13.8)	10 (16.9)	2 (7.1)	0.323
PCI success (%)	77 (96.3)	55 (96.5)	22 (95.7)	0.858
CABG (%)	2 (2.3)	0 (0%)	2 (7.1)	0.091
Number of treated vessels	1.26 ± 0.54	1.26 ± 0.48	1.25 ± 0.68	0.932
Time to reperfusion (hours)	6.1 ± 5.2	5.6 ± 4.6	7.3 ± 5.9	0.072
Duration of IABP support (hours)	54.5 ± 24.0	55.4 ± 20.8	52.1 ± 31.3	0.654
TIMI flow before PCI	0.51 ± 0.67	0.51 ± 0.67	0.50 ± 0.71	0.958
TIMI flow after PCI	2.77 ± 0.57	2.82 ± 0.51	2.67 ± 0.70	0.343
CPR periinterventional (%)	12 (13.8)	5 (8.5)	7 (25.0)	0.040

Values are expressed as *n *(%), or mean ± SD. CABG, coronary artery bypass graft; CPR, cardiopulmonary resuscitation; EF, ejection fraction; IABP, intraaortic balloon counterpulsation; PCI, percutaneous coronary intervention; TIMI, thrombolysis in myocardial infarction.

### Hemodynamic parameters and catecholamine support

Values of hemodynamic measurements at admission are illustrated in Table 3. For CPI, a significant difference was observed. In addition, MAP and systolic BP were reduced within the nonsurvivor group, whereas heart rate and maximal CVP within the first 24 hours were elevated in this group. Patients (68; 78.2%) required supportive catecholamine therapy within the first 24 hours, the more in the nonsurvivor group (Table 3).

**Table 3:** Hemodynamics and catecholamine support according to outcome

	All patients(*n *= 87)	Survivors(*n *= 59)	Nonsurvivors(*n *= 28)	*P *value
Hemodynamics				
CI (L/min/m^2^)	2.15 ± 0.83	2.16 ± 0.78	2.12 ± 0.93	0.825
CPI (W/m^2^)	0.35 ± 0.15	0.37 ± 0.15	0.30 ± 0.15	0.041
CPO (W)	0.67 ± 0.26	0.70 ± 0.27	0.60 ± 0.25	0.102
Microcirculatory PI	97.51 ± 46.17	101.74 ± 43.08	86.23 ± 53.22	0.181
SV (ml)	52.6 ± 16.5	54.7 ± 16.8	48.1 ± 15.4	0.078
SVI (ml/m^2^)	26.8 ± 9.4	28.0 ± 9.0	24.3 ± 9.8	0.105
SW (g m)	37.5 ± 18.5	39.4 ± 18.6	31.7 ± 17.5	0.121
SWI (g m/m^2^)	19.1 ± 10.2	20.2 ± 10.1	16.0 ± 10.2	0.137
LVW (kg m/min)	2.88 ± 1.35	2.97 ± 1.35	2.60 ± 1.37	0.327
LVWI (kg m/min/m^2^)	1.47 ± 0.77	1.53 ± 0.74	1.30 ± 0.84	0.320
SVR (dynes sec/cm^5^)	1,251.8 ± 672.9	1,285.3 ± 705.6	1,146.6 ± 566.9	0.444
SVRI (dynes sec/cm^5^/m^2^)	2,281.6 ± 898.2	2,302.9 ± 894.2	2,216.2 ± 938.8	0.756
MAP (mm Hg)	71.1 ± 15.0	74.8 ± 13.7	63.8 ± 14.9	0.002
Min. MAP (mm Hg) day 1	55.1 ± 13.1	58.9 ± 10.9	45.8 ± 13.7	0.001
Systolic BP (mm Hg)	94.8 ± 22.7	98.6 ± 21.7	85.5 ± 23.0	0.036
Min. systolic BP (mm Hg) day 1	70.9 ± 17.7	75.8 ± 15.7	59.0 ± 16.8	0.001
LVEDP (mm Hg)	23.1 ± 8.9	22.9 ± 9.3	23.7 ± 8.1	0.748
CVP (mm Hg)	12.7 ± 5.8	12.2 ± 5.0	13.8 ± 7.6	0.416
Max. CVP (mm Hg) day 1	19.0 ± 8.3	17.2 ± 7.4	23.9 ± 8.9	0.008
HR (beats/min)	82.1 ± 21.2	78.7 ± 19.4	89.4 ± 23.2	0.043
Min. HR (beats/min) day 1	59.0 ± 17.3	56.4 ± 13.7	65.5 ± 23.3	0.115
Max. HR (beats/m) day 1	123.2 ± 25.3	118.8 ± 23.8	134.0 ± 26.2	0.032
Scvo_2 _(%)	66.3 ± 16.9	67.6 ± 16.5	63.2 ± 18.0	0.378
Min. Scvo_2 _(%) day 1	56.3 ± 13.5	56.2 ± 12.4	56.7 ± 16.3	0.905
Inotropic and vasopressor support				
Catecholamine use (%)	68 (78.2%)	43 (72.9)	25 (89.3)	0.101
Dobutamine use (%)	52 (59.8)	33 (55.9)	20 (71.4)	0.240
Mean dose of dobutamine (µg/min) day 1	500 (0/975)	250 (0/700)	950 (0/1,075)	0.021
Norepinephrine use (%)	64 (73.6)	39 (66.1)	25 (89.3)	0.036
Mean dose of nor-epinephrine (µg/min) day 1	10 (0/61)	3 (0/15)	100 (40/130)	<0.0001
Epinephrine use (%)	25 (28.7)	8 (13.6)	18 (64.3)	<0.0001
Mean dose of epinephrine (µg/min) day 1	0 (0/0.5)	0 (0/0)	15 (0/80)	<0.0001
Total number of catecholamines	2.0 (1.0/2.0)	2.0 (0.0/2.0)	3.0 (2.0/3.0)	0.001
Total mean dose of catecholamines (µg/min) day 1	503 (0.6/1013)	305 (0/706)	995 (165/1310)	0.006

Values are expressed as *n *(%), mean ± SD, or median with interquartile range (quartile 1 to quartile 3). Beats/min, beats per minute; BP, blood pressure; CI, cardiac index; CO, cardiac output; CPI, cardiac power index; CPO, cardiac power output; CVP, central venous pressure; HR, heart rate; LVEDP, left ventricular end-diastolic pressure; LVW, left ventricular work; LVWI, left ventricular work index; MAP, mean arterial pressure; max, maximal; min, minimal; Scvo_2_, central venous oxygen saturation; SV, stroke volume; SVI, stroke volume index; SVR, systemic vascular resistance; SVRI, systemic ventricular resistance index; SW, stroke work; SWI, stroke work index.

### Predictive value of Nt-proBNP, IL-6, and PCT for 30-day mortality

**Figure 1:**
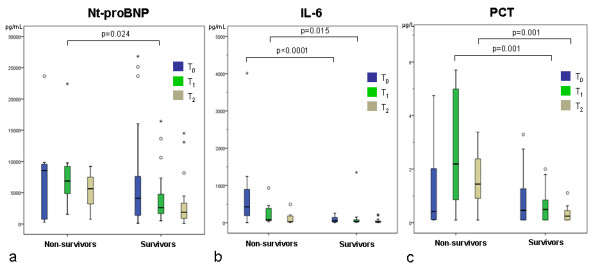
**Serial measurements of Nt-proBNP (a), interleukin-6 (IL-6) (b) and procalcitonin (PCT) (c) in survivors versus nonsurvivors at admission (T_0_), after 24 hours (T_1_), and after 72 hours (T_2_)**. In the box-and-whisker plot, the central box represents the interquartile range (IQR); the middle line represents the median. Outside values (○) are smaller/larger than the lower/upper quartile ± 1.5 × the IQR; far-out values (*) are smaller/larger than the lower/upper quartile ± 3 × the IQR.

Main purpose of the present study was to determine the predictive value of serial measurements of Nt-proBNP, IL-6, and PCT for 30-day mortality. Medians (interquartile ranges) in all patients accounted for Nt-proBNP, 4,065 (1,147/9,775) pg/ml at T_0_, 3,588 (1,667/7,376) pg/ml at T_1_, and 2,409 (1,082/5,235) pg/ml at T_2_; IL-6, 74.7 (18.1/255.5) pg/ml at T_0_, 46.5 (28.2/136.0) pg/ml at T_1_, and 26.8 (10.4/83.4) pg/ml at T_2_; PCT, 0.25 (0.10/1.40) µg/L at T_0_, 0.53 (0.10/1.68) µg/L at T_1_, and 0.43 (0.15/0.90) µg/L at T_2_. Significant differences between survivors and nonsurvivors were seen for Nt-proBNP at T_1 _(2,831 versus 6,871 pg/ml; *P *= 0.024), for IL-6 at T_0 _(30.7 versus 424.0 pg/ml; *P *< 0.0001), and T_1 _(41.9 versus 79.1 pg/ml; *P *= 0.015), and for PCT at T_1 _(0.46 versus 2.42 µg/L; p = 0.001) and T_2 _(0.32 versus 1.44 µg/L; *P *= 0.001) (Figure 1). In contrast, for CRP, no statistical differences were seen (data not shown).

**Figure 2:**
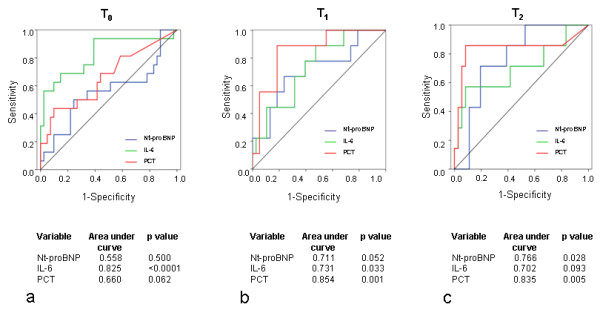
**Receiver operating characteristic (ROC) curves for 30-day mortality**. Analysis of 30-day mortality calculated from values of Nt-proBNP, interleukin-6 (IL-6), and procalcitonin (PCT) at admission (T_0_) **(a)**, after 24 hours (T_1_) **(b)**, and after 72 hours (T_2_) **(c)**. ROC curve analysis with area under the curve and significance levels.

**Figure 3:**
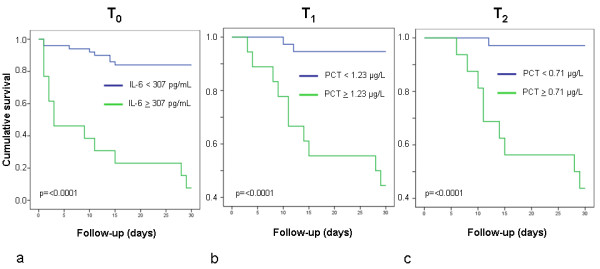
**Kaplan-Meier survival curves according to cut-off levels of interleukin-6 (IL-6) (a) and procalcitonin (PCT) (b, c)**. Estimated 30-day survival in patients with levels of IL-6 above or below the cut-off level (307 pg/ml) at admission (T_0_) (a); of PCT (1.23 µg/L) after 24 hours (T_1_) (b); of PCT (0.71 µg/L) after 72 hours (T_2_) (c). *P *values were estimated by log-rank test.

ROC curves calculated for Nt-proBNP, IL-6, and PCT at T_0_, T_1_, and T_2 _are illustrated in Figure 2. At the earliest point in time, T_0_, only IL-6 was predictive of 30-day mortality; at T_1_, IL-6 and PCT showed significant *P *values, at T_2 _PCT and Nt-proBNP were predictive of mortality. Comparing the area under the curve of the parameters at each point in time, the highest accuracy in predicting 30-day mortality was seen for IL-6 at T_0_, for PCT at T_1_, and for PCT at T_2 _(Figure 2). Notably, the highest level of significance was found for IL-6 at T_0_. Based on these analyses, corresponding cut-off levels with highest sensitivity and specificity were calculated: T_0 _IL-6, 307 pg/ml (sensitivity, 57.1%; specificity, 97.7%); T_1 _PCT, 1.23 µg/L (sensitivity, 83.3%; specificity, 81.4%); T_2 _PCT, 0.71 µg/L (sensitivity, 90.0%; specificity, 82.9%). Based on these cut-off levels, 30-day survival rates of patients according to Kaplan-Meier analyses differed highly significantly (Figure 3). Survival of patients with IL-6 levels below the cut-off value was significantly improved compared with that of patients with IL-6 levels above the cut-off value, irrespective of the presence of SIRS (*P *< 0.05).

### Organ failure as predictor of 30-day mortality

Acute kidney injury (AKI) occurred in 31 (35.6%) patients. AKI appeared significantly more frequent in nonsurvivors (67.9%) compared with survivors (20.7%; *P *< 0.0001). Serum creatinine at admission (1.99 (1.45/3.45) mg/dl versus 1.34 (1.09/1.58) mg/dl) and eGFR (38.2 ± 19.4 ml/min versus 67.7 ± 31.5 ml/min) after the first 24 hours also differed significantly between the groups (*P *< 0.0001). The incidence of MOF was increased dramatically in the nonsurvivor group (60.7% versus 6.8%; *P *< 0.0001). According to Kaplan-Meier analyses, the overall 30-day survival rate of patients with MOF was markedly lower than that of patients without MOF (15.0% versus 84.8%; *P *< 0.0001 by log-rank test). The overall survival rate of patients with an APACHE II score above the median was in trend lower than that of patients below this cutoff (62.9% versus 81.8%; *P *= 0.065). Initial serum lactate as a marker for organ hypoperfusion was clearly elevated within the nonsurvivor group (5.8 (1.6/12.7) m*M *versus 1.4 (1.1/3.2) m*M*; *P *< 0.01).

### Univariate and multivariate analyses of predictors for 30-day mortality

In univariate analysis, significant values were found for Nt-proBNP at T_1_, for IL-6 at all points in time, and for PCT also at all points in time (Table 4). Further significant predictors are shown in Table 4. Within the multivariate analysis including age, CPI at admission, total mean dose of catecholamines within first 24 hours, lactate at admission, creatinine at admission, IL-6 at admission, and PCT at admission, age, creatinine, and IL-6 were significant determinants of 30-day mortality in which IL-6 showed the highest level of significance (Table 5).

**Table 4:** Univariate analysis predicting 30-day mortality

	HR (95% CI)	*P *value
Patient characteristics		
Age, per 10 years	1.638 (1.174-2.285)	0.004
EF, per 5%	1.061 (0.887-1.269)	0.516
Left main	2.143 (0.504-9.117)	0.302
STEMI	1.005 (0.479-2.108)	0.989
Mechanical ventilation	1.313 (0.625-2.762)	0.472
Hemodynamics and catecholamines		
CO, L/min	0.986 (0.760-1.279)	0.915
CI, L/min/m^2^	0.934 (0.594-1.468)	0.768
CPI, 0.1 W/m^2^	0.766 (0.593-0.991)	0.043
CPO, 0.1 W	0.874 (0.747-1.023)	0.094
Microcirculatory PI/10	0.888 (0.795-0.992)	0.036
SVRI, 100 dynes sec/cm^5^/m^2^	0.992 (0.937-1.050)	0.776
MAP, 5 mm Hg	0.821 (90.726-0.928)	0.002
Heart rate, 5 beats/min	1.086 (1.003-1.176)	0.041
Catecholamine use	3.073 (0.716-13.198)	0.131
Total number of catecholamines	2.332 (1.350-4.029)	0.002
Mean dose of catecholamines, 10 µg/min	1.014 (1.004-1.024)	0.006
Organ failure		
Acute kidney injury	5.084 (2.290-11.290)	<0.0001
SIRS	1,412 (0.639-3.121)	0.394
MOF	8.317 (3.754-18.425)	<0.0001
Lactate, m*M*	1.164 (1.086-1.247)	<0.0001
APACHE II > Median	2.390 (0.908-6.294)	0.078
Laboratory measurements		
Creatinine T_0_, mg/dl	1.299 (1.092-1.546)	0.003
Creatinine T_1_, mg/dl	2.155 (1.534-3.028)	<0.0001
Creatinine T_2_, mg/dl	1.562 (0.626-3.901)	0.339
Nt-proBNP T_0_, 1,000 pg/ml	1.022 (0.988-1.056)	0.204
Nt-proBNP T_1_, 1,000 pg/ml	1.111 (1.045-1.182)	0.001
Nt-proBNP T_2_, 1,000 pg/ml	1.109 (0.959-1.282)	0.161
IL-6 T_0_, 100 pg/ml	1.019 (1.008/1.030)	0.001
IL-6 T_1_, 100 pg/ml	1.134 (1.023-1.257)	0.016
Il-6 T_2_, 100 pg/ml	1.550 (1.031-2.331)	0.035
PCT T_0_, µg/L	1.368 (1.075-1.741)	0.011
PCT T_1_, µg/L	1.266 (1.122-1.429)	<0.0001
PCT T_2_, µg/L	2.980 (1.687-5.265)	<0.0001

IL-6, interleukin-6; MOF, multiorgan failure; Nt-proBNP, N-terminal pro-B-type natriuretic peptide; PCT, procalcitonin; SIRS, systemic inflammatory response syndrome; other abbreviations as in Tables 1 through 3.

**Table 5:** Multivariate Cox proportional hazard ratios for 30-day mortality

	Hazard ratios (95% CI)	*P *value
Age, per 10 years	3.372 (1.548-7.348)	0.002
CPI, 0.1 W/m^2^	0.790 (0.506-1.231)	0.297
Mean dose of catecholamines, 10 µg/min	1.013 (0.997-1.028)	0.116
Lactate T_0_, m*M*	1.007 (0.839-1.209)	0.939
Creatinine T_0_, mg/dl	1.589 (1.033-2.444)	0.035
IL-6 T_0_, 100 pg/ml	1.036 (1.017-1.055)	<0.0001
PCT T_0_, µg/L	1.236 (0.843-1.812)	0.277

Abbreviations as in Tables 1 through 4.

## Discussion

The aim of the present study was to analyze the predictive value of a broad spectrum of clinical and laboratory parameters in patients with CS due to AMI treated with immediate revascularization. To the best of our knowledge, this is the first prospective investigation in the early phase of AMI and CS analyzing serial measurements of Nt-proBNP, IL-6, and PCT as predictors of 30-day mortality.

Growing evidence indicates that an aggressive strategy based on immediate coronary revascularization and IABP support is the most effective therapy for CS due to AMI [2,5]. Characteristics of our study population were similar to those of the patients undergoing revascularization in the Should We Emergently Revascularize Occluded Coronaries for Cardiogenic Shock (SHOCK) trial [15]. The present study was based on a therapy strategy that included urgent revascularization and support by an IABP. In contrast to the SHOCK trial, the predominant proportion of patients was revascularized by PCI with a high success rate and revealed a 30-day mortality of 32%, an incidence lower than that reported in the SHOCK trial [15].

### Nt-proBNP, IL-6, and PCT as predictors of outcome

The measurement of serum levels of natriuretic peptides is established in the diagnosis and follow-up of chronic HF. Maisel *et al*. [6] showed that BNP may be helpful in diagnosing acute HF in the emergency setting. Moreover, Nt-proBNP has been suggested as a useful marker of high risk in acute myocardial infarction. Higher baseline levels of BNP in patients presenting with STEMI were associated with impairment of reperfusion after fibrinolytic therapy, whereas patients in the highest BNP-concentration quartile had an 11-fold risk of dying by 30 days [16]. Similarly, Nt-proBNP was previously shown to be predictive of myocardial damage, development of CS, and short- and long-term mortality [17,18]. Data concerning the predictive value of serial Nt-proBNP measurements in patients with manifest CS due to AMI are rare. In the present study, the survivor group showed a decline of Nt-proBNP levels within 72 hours, whereas in the nonsurvivor group, maximum levels were reached after 24 hours. Significant differences of Nt-proBNP were found only after 24 hours between survivors and nonsurvivors. Notably, Nt-proBNP revealed no predictive value for the development of MOF. In a retrospective study with 58 patients with CS largely secondary to AMI, initial Nt-proBNP levels above the median were associated with worsened outcome [19]. However, Nt-proBNP levels were not analyzed serially. Next to the retrospective study design, the number of patients was small in particular if the cohort was further subdivided. Among the 47 patients who had AMI as the primary cause of CS, only in 33 patients was coronary revascularization performed, with a success rate of 85% [19]. In our study, revascularization was attempted in 94% of patients, with a success rate of 96%, and an IABP was placed in all patients.

In patients with septic shock, IL-6 levels have been shown to correlate with disease severity and outcome [20]. However, elevations are not sepsis specific. It has been shown that patients with CS and manifest MOF exhibit similarly high IL-6 levels, as in patients with septic shock [10]. In the setting of AMI, IL-6 was identified as independent prognostic marker [21]. In the present study, IL-6 levels differed significantly between the survivor and nonsurvivor groups at the early points in time, in which the values at admission showed an impressive discrepancy. In accordance with that, univariate analysis confirmed the predictive value of IL-6 for 30-day mortality at the early points in time. IL-6 levels at admission revealed the highest level of significance within the ROC analyses. A cut-off level of 307 pg/ml showed a very clear differentiation between survivors and nonsurvivors within the Kaplan-Meier curves, associated with a very high specificity of 98%. Moreover, in contrast to Nt-proBNP, IL-6 levels at T_0 _and T_1 _were predictive for occurrence of MOF. Our data confirm the results of a small retrospective study with only initial measurements of IL-6 that also showed an independent predictive value of IL-6 for 30-day mortality [11]. It is hypothesized that next to ischemic myocardium, whole body ischemia, endotoxin translocation from the gut, excessive vasopressor therapy, and noncardiac organ failure are also contributing factors to IL-6 release [22]. Possibly, IL-6 constitutes not only an innocent bystander of inflammatory activation, but also might be an aggravating factor because IL-6 determines negative inotropic effects on the myocardium [23].

Several studies have demonstrated that elevated levels of PCT indicate bacterial infection accompanied by a systemic inflammatory reaction [12]. Nevertheless, PCT elevation has been documented in infection-independent systemic inflammatory reactions. In recent studies, PCT levels were found to correlate with the extent of coronary artery disease and adverse outcome [24]. In a retrospective study, CS patients showed high PCT concentrations, especially in the presence of MOF and in the absence of signs of infection [25]. In a further investigation, PCT values were significantly higher in CS patients compared with patients with uncomplicated AMI [26]. The prognostic relevance of PCT in CS was not evaluated. In our study, both survivors and nonsurvivors reached maximum levels of PCT at T_1_. Significant differences between the two groups were seen at T_1 _and T_2 _and were strongly associated with outcome. ROC analysis showed the highest accuracy of predicting 30-day mortality for PCT at T_1 _and T_2 _with a sensitivity and specificity between 80% and 90%. Notably, when comparing the area under the curve of Nt-proBNP, IL-6, and PCT, at all points in time, PCT at T_1 _showed the highest value.

### Hemodynamics and organ failure as predictors of outcome

Hemodynamic data, including CO and pulmonary wedge pressure, were shown to be the strongest predictors of death in the GUSTO CS trial [27]. In a retrospective analysis from the SHOCK trial registry, CPI was the strongest predictor of mortality [28]. In our study, hemodynamic measurements also showed a predictive value of CPI in the univariate but not multivariate analysis.

Impaired renal function is one of the strongest risk factors for cardiovascular mortality. In a retrospective analysis in patients with CS due to AMI, 33% developed an AKI within 24 hours of shock onset and showed a significantly higher mortality rate than did patients without AKI [29]. In a recent prospective study of STEMI patients with CS, AKI occurred in 55% of patients, and the in-hospital mortality rate was significantly higher in patients developing AKI than in patients without AKI [30]. In our study with STEMI and also NSTEMI patients, AKI occurred in 36% of patients during first 72 hours after admission. All measured parameters of renal insufficiency were clearly associated with worse outcome.

Irreversible MOF is the main cause of death in CS. Until now, only a few studies addressed the issue of MOF in patients with CS. In a small study, 35% of patients with CS exhibited MOF at admission categorized by the SOFA score, and an additional 20% developed MOF later during the clinical stay [10]. In our study, 24% of patients fulfilled criteria of MOF with a highly significant difference to the disadvantage of nonsurvivors.

### IL-6 is the strongest independent predictor of 30-day mortality

As the central finding of our study, multivariate analysis including age, creatinine, lactate, CPI, mean dose of catecholamines, and IL-6 or PCT showed independent predictive values for age, creatinine, and IL-6, in which IL-6 reached the highest level of significance. Therefore, IL-6 might be a useful marker to identify very early CS patients who are at high risk of worse outcome. In contrast, the relevance of Nt-proBNP as an early prognostic marker must be challenged. PCT showed also a highly significant impact but primarily after the very early phase of AMI and CS.

## Conclusions

The present study demonstrates that in patients with AMI and CS treated with immediate revascularization and IABP, the destination of IL-6 at admission reliably predicts 30-day mortality. For clinical practice, it is noticeable that a cut-off level of 307 pg/ml is associated with a very high specificity but moderate sensitivity. In contrast, Nt-proBNP seemed to be of lower relevance.

In conclusion, selective measurements of circulating inflammatory markers allow early prognostic estimation of patients with CS due to AMI. Potential therapeutic and prognostic interventions modulating the systemic inflammatory reaction have to be analyzed in further studies.

## Key messages

• Systemic inflammation is observed in many patients with cardiogenic shock due to myocardial infarction.

• Interleukin-6 represents a reliable independent very early prognostic marker of 30-day mortality with high specificity.

• Procalcitonin is a very reliable predictor of 30-day mortality 24 and 72 hours after admission, with high sensitivity and specificity.

## Abbreviations

AMI: acute myocardial infarction; CABG: coronary artery bypass graft; CPR: cardiopulmonary resuscitation; CS: cardiogenic shock; IL-6: interleukin-6; MOF: multiorgan failure; NSTEMI: non-ST-elevation myocardial infarction; Nt-proBNP: N-terminal pro-B-type natriuretic peptide; PCI: percutaneous coronary intervention; PCT: procalcitonin; SIRS: systemic inflammatory response syndrome; STEMI: ST-elevation myocardial infarction.

## Competing interests

The authors declare that they have no competing interests.

## Authors' contributions

RPA, UMB, GN, and JOS participated in the conception and design or analysis and interpretation of data. RPA, UMB, RF, VT, JWS, GN, and JOS drafted the manuscript or revised it critically for important intellectual content. RPA, UMB, GN, and JOS gave final approval of the manuscript. All authors read and approved the manuscript for publication.
